# Trends in undernutrition mortality among children under five years of age and adults over 60

**DOI:** 10.7705/biomedica.5937

**Published:** 2022-03-01

**Authors:** Roxanna Uribe-Quintero, Luz Stella Álvarez-Castaño, Beatriz Caicedo-Velásquez, Isabel Cristina Ruiz-Buitrago

**Affiliations:** 1 Escuela de Nutrición y Dietética, Universidad de Antioquia, Medellín, Colombia Universidad de Antioquia Escuela de Nutrición y Dietética Universidad de Antioquia Medellín Colombia; 2 Facultad Nacional de Salud Pública, Universidad de Antioquia, Medellín, Colombia Universidad de Antioquia Facultad Nacional de Salud Pública Universidad de Antioquia Medellín Colombia; 3 Escuela de Graduados, Universidad CES, Medellín, Colombia Universidad CES Escuela de Graduados Universidad CES Medellín Colombia; 4 Facultad de Ciencias Sociales, Universidad Externado de Colombia, Bogotá, D.C. Colombia Universidad Externado de Colombia Facultad de Ciencias Sociales Universidad Externado de Colombia Bogotá, D.C. Colombia

**Keywords:** Undernutrition, infant mortality, spatial analysis, health status disparities, social determinants of health, desnutrición, mortalidad infantil, análisis espacial, disparidades en el estado de salud, determinantes sociales de la salud

## Abstract

**Introduction:**

Children under five years of age living in poor areas and with low availability of healthy food have a higher risk of undernutrition-related mortality. However, this relationship has not been well established among older adults.

**Objective:**

To analyse socioeconomic inequality trends related to undernutrition mortality in children under five years of age and adults over 60 in Colombian municipalities during 2003-2009 and 2010-2016.

**Materials and methods:**

We conducted an ecological study of trends between 2003 and 2016. The study population consisted of children under five years of age and adults over 60 residing in the Colombian municipalities during the study period. We estimated smoothed and standardized mortality rates by fitting a hierarchical Bayesian model and explored their relationship with five socioeconomic area-level variables.

**Results:**

In most of the municipalities, undernutrition-related mortality was three times higher in older adults compared to children. Moreover, the difference in the risk of undernutrition-related mortality between municipalities showed a marked reduction. Finally, the poor and less developed municipalities had higher rates of undernutrition-related mortality in children; conversely, wealthier territories had higher rates in older adults.

**Conclusions:**

Although in most of the municipalities the mortality rates due to undernutrition in children under five and older adults have decreased, their socioeconomic conditions influence in different ways the risk of mortality for these two populations so there is the need to develop age-specific strategies to close social gaps considering the structural conditions of the areas.

## Resumen

**Introducción.:**

Los niños menores de cinco años que viven en zonas pobres y con poca disponibilidad de alimentos saludables, tienen mayor riesgo de mortalidad relacionada con la desnutrición, relación que no se ha determinado claramente entre los adultos mayores.

**Objetivo.:**

Analizar las tendencias de la desigualdad socioeconómica relacionada con la mortalidad por desnutrición en niños menores de cinco años y adultos mayores de 60 en los municipios colombianos durante 2003-2009 y 2010-2016.

**Materiales y métodos.:**

Se hizo un estudio ecológico de tendencias entre 2003 y 2016. La población de estudio incluyó a niños menores de cinco años y adultos mayores de 60 residentes en los municipios colombianos durante el período de estudio. Se estimaron las

tasas de mortalidad suavizadas y estandarizadas ajustadas mediante un modelo bayesiano jerárquico y se exploró su relación con cinco variables socioeconómicas a nivel de área.

**Resultados.:**

En la mayoría de los municipios, la mortalidad relacionada con la desnutrición fue tres veces mayor en los adultos mayores que en los niños. Además, la diferencia en el riesgo de mortalidad por desnutrición entre municipios mostró una marcada reducción. Por último, los municipios pobres y menos desarrollados registraron tasas más altas de mortalidad infantil relacionada con la desnutrición, en tanto que, en los territorios más ricos, las tasas más altas se registraron en adultos mayores.

**Conclusiones.:**

Si bien en la mayoría de los municipios las tasas de mortalidad por desnutrición en niños menores de cinco años y adultos mayores han disminuido, sus condiciones socioeconómicas influyen de diferente manera en el riesgo de mortalidad de estas dos poblaciones, por lo que surge la necesidad de desarrollar estrategias específicas por edades para cerrar las brechas sociales con base en las condiciones estructurales de las zonas.

Historically, undernutrition primarily affected children under five years of age but today its influence on the health condition of older adults is more widely known ^1^. Undernutrition is the pathological state resulting from inadequate consumption of one or more essential nutrients. It involves weight loss associated with caloric and protein deficits and low levels of other specific nutrients that are necessary for the adequate homeostasis of the organism ^2^. In the case of children, those who are affected have limitations in their physical, mental, and psychomotor development. They can suffer biochemical and physiological disorders such as growth delays, cognitive impairments, and lower physical and intellectual capacity ^3^. In older adults, undernutrition increases the risk of mortality, hospitalization, hip fractures, and institutionalization due to the reduction of functional autonomy ^1^.

The Colombian Health Situation Analysis (HSA) surveillance system ^4^ shows that the country had a decreasing trend in undernutrition-related mortality rates in children under five, from 14.87 per 100,000 children under five in 2005 to 6.82 deaths in 2014. In the case of older adults, a study by Cardona, *et al.*^5^ found that in 2008 in Colombia, the mortality rate from nutritional deficiency in those over 65 years was 34.5 deaths per 100,000. The same study also highlighted the great variability within the country in mortality risk: with the highest rate (550.5 deaths per 100,000 people over 65 years) in the department of Vaupés and the lowest (13.5 deaths per 100,000 people over 65 years) in Tolima ^5^. Other departments that exceeded the national rate were Guaviare, Guainía, Bolívar, and Atlántico.

Regarding the determinants of undernutrition-related mortality, one of the most important factors is families poverty level, as their financial restrictions mean limited access to healthy food ^6^, while among older adults, it is associated with living alone, deficient social support networks, and limited functional ability ^7^. Several studies have emphasized the relationship between undernutrition and the place of residence showing that children and older adults living in poor areas have a higher risk of undernutrition ^6^^,^^8^, as do those living in areas with low availability of healthy food ^9^. Additionally, there are other factors associated with undernutrition, especially in children, such as unfavorable socioeconomic conditions related to inadequate sanitation ^10^, food, and health care practices ^11^^,^^12^.

In Colombia, poverty and development levels in municipalities are very heterogeneous and, therefore, we can assume that these differing conditions contribute to an unequal distribution of undernutrition-related mortality. In such context, our study focused on establishing if the differences between municipalities also reflected on their undernutrition-related mortality rates in children under five years of age and adults over 60 between 2003 and 2016. Additionally, we aimed to determine the relationship of those mortality rates with the socioeconomic conditions of the municipalities.

## Materials and methods

We conducted a longitudinal ecological study to analyze aggregated data of Colombian residents under five and over 60 years of age between 2003 and 2016. The geographic units of analysis were: six regions, 18 subregions, and 1,096 municipalities.

**Figure 1:**
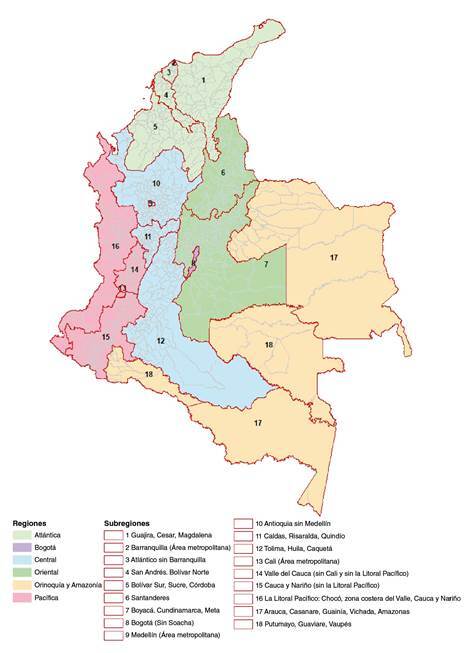
Administrative levels in Colombia: Regions (in colors), subregions (numbered), and municipalities (light gray), Colombia, 2005

To improve municipal estimates, we created a geographic map at this level where the non-municipalized areas were added to their respective cities. Likewise, the four municipalities created after 2005 were regrouped into their original municipalities. Additionally, the municipalities of the *Archipiélago de San Andrés, Providencia and Santa Catalina* were excluded because they did not have contiguous or neighboring geographic areas allowing reliable municipal estimates of their mortality risks. Consequently, we analyzed data from 1,096 municipalities (figure 1).

### Information sources

We obtained the data on deaths from the vital statistics available on the National Administrative Department of Statistics website ^13^ and aggregated them according to the geographic units of analysis. The data regarding municipal socioeconomic characteristics were obtained from the *Sistema Nacional de Vigilancia en Salud Pública* (SIVIGILA), the United Nations Development Programme (UNDP), and the *Departamento Nacional de Planeación* (DNP).

### Variables

*Dependent variables.* We analyzed two aggregated variables: 1) The municipal undernutrition mortality rate in children under five per 100,000 children under five, and 2) the municipal undernutrition mortality rate in people over 60 per 100,000 inhabitants over 60 years of age.

Undernutrition deaths were defined as those with primary cause codes according to the 10th Revision of the International Classification of Diseases corresponding to undernutrition (E40-to-E46), other nutritional deficiencies (E50.0-to-E64.9), or nutritional anemia (D50.0-to-D53.9).

*Independent variables.* These included the following municipal socioeconomic conditions:

- The percentage of the population with unsatisfied basic needs (UBN) was measured in 2005 to characterize poverty in municipalities by quantifying the proportion of people experiencing at least one of the following conditions: overcrowding, inadequate housing, inadequate water supply, lack of sewers, and poor school attendance ^14^. Municipalities were categorized into quintiles, the lowest one comprising the wealthiest municipalities and the highest one, the poorest.
- The human development index (HDIm) measured in 2005 represents the degree of development achieved by a municipality in terms of quality of life, education, and per capita gross domestic product (GDP) ^15^. Municipalities were categorized into quintiles, the lowest one including those with low human development and the highest one, those with very high human development.
- The multidimensional poverty index (MPI) identifies multiple deficiencies in the areas of health, education, and living standards in the period 2005-2014. Municipalities were categorized into quintiles, the lowest one including the richest group of municipalities and the highest one, the poorest municipalities ^16^.
- The institutional capacity of the municipal government (*índice de desempeño integral*), an index that sorted and compared municipalities between 2006 and 2015 based on the following aspects: effectiveness in meeting the goals of their development plans, efficiency in the provision of basic services, compliance with budget execution requirements defined by law, and administrative and fiscal management. The municipalities were classified into four performance groups: outstanding or satisfactory, medium, low, and critical ^17^.
- The dynamics of the index of water quality for human consumption (*índice de riesgo de la calidad del agua,* IRCA) 2007-2010, which measures the water quality-related risk ^18^ and classifies municipalities into five water-quality risk groups: none, low, medium, high, and unviable sanitary risk. Given the variability of the index during the available period (2007, 2008, 2009, and 2010), its dynamics pattern was summarized into one variable for the present analysis. The resulting variable classifies the municipalities into five groups: improving (municipalities with constant changes toward the no-risk category), constant positive (municipalities oscillating between the no-risk or low- risk categories), constant negative (municipalities oscillating between the low, medium, high, or unviable sanitary risk categories), and worsening (municipalities with constant changes towards the unviable sanitary risk category).

### Data processing

For the descriptive analysis, we estimated national and regional mortality age-specific crude rates for two periods: 2003-2009 and 2010-2016 using the total number of undernutrition deaths (under five years old or over 60 years old) as the numerator. The denominator was either the total population of children under five or people over 60 multiplied by 100,000.

As for the municipal rate estimates, we used the Bayesian hierarchical model proposed by Besag, *et al.* (BYM), to obtain smoothed rates for the whole period: 2003-2016 ^19^, which improved the precision of the estimated rates, especially for those municipalities with a low number of deaths due to undernutrition and/or a small population. In brief, this model combined the aggregated number of deaths in each municipality with the average of the neighboring municipalities, thus decreasing the variability of the estimates. The BYM model was specified as follows:




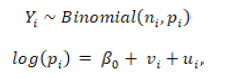




where *n*_*i*_ is the denominator in each municipality and the rate in each municipality, *i,* is represented by *p*_*i*_ The model has two random effects that represent non-spatial and spatial variability, *v*_*i*_ *v*_*i*_ , and *u*_*i*_ *u*_*i*_ . These effects allow for the estimation of the smoothed rate for each municipality with the equation: *exp(β0 +v*_*i*_ *+u*_*i*_ *)*. The *a priori* distributions of spatial effects was assigned through an intrinsic conditional autoregressive (ICAR) distribution with variance σ^*2*^ while for the non-spatial effects we used a normal distribution with zero as mean and variance σ_*v*_^*2* (^^19^. A half-normal distribution with a mean of 0 and a precision of 0.0001 was assigned to the standard deviations σ_*v*_ and σ_*u*_^20^. For parameter ∝∝, we assigned a normal “vague prior” distribution.

The model estimated the smoothed mortality rate (SMR) with the corresponding 95% confidence interval for each municipality, as well as the *a posteriori* probability (PrP), which indicates whether each municipality had a smoothed rate significantly higher than the rate of Colombia (p<0.05). This was calculated as *PrP*_*i*_ *= Probability (smothed P*_*i*_ *> rate in Colombia)*The model was estimated by using the integrated nested Laplace approximation (INLA) method available in the statistical package R.2.15.3 INLA library ^21^^,^^22^.

### Statistical analysis

Tables and maps were used for the descriptive analysis of regional and municipal estimates by period and age-group. We extended the ecological Bayesian hierarchical model at level two to to include socioeconomic (SE) variables and analyze their relationship with municipal smoothed rates. NBI, HDIm, and MPI were categorized into quintiles and included in separate models as four dummy variables (QSE) with the less poor or less developed areas serving as the reference group. For the other two socioeconomic variables, outstanding performance and risk-free water quality were used as reference:




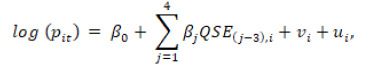




where each municipality is represented as *i and β*_*j*_ is the effect of the category of the socioeconomic variable on the smoothed rates. By using exp *[β*_*1*_ *] (j=1,...,4)*, the results of the models are presented as the relative risk (RR) of mortality in each socioeconomic group along with the corresponding 95% confidence intervals (95% CI).

The research protocol was approved by *Universidad de Antioquia* Ethics Committee for the health area (reference number 161, 09/March/2017).

## Results

Between 2003 and 2016 a total of 2,754,943 deaths were reported in Colombia and 24,388 of them were due to undernutrition, i.e., a rate for the period of 3.85 per 100,000 inhabitants. According to the age group, 23.8% (n=5,804) of the total mortality due to undernutrition corresponded to children under five and 61.4% (n=14,974) to people over 60. The estimated rate during the period was 8.6 deaths per 100,000 children under five and 24.5 deaths per 100,000 adults over 60.

When we compared the two periods (2003-2009 vs 2010-2016), we found a general decrease in mortality rates: from 10.0 to 7.2 deaths per 100,000 children under five and from 26.0 to 23.2 deaths per 100,000 people over 60.

**Figure 2:**
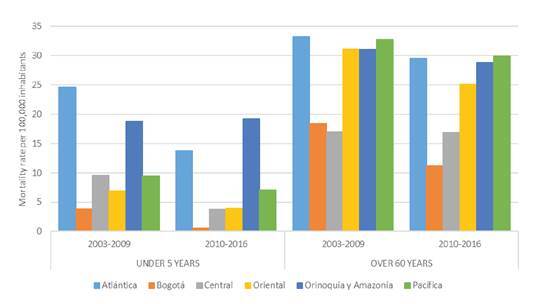
Mortality rate per 100,000 inhabitants due to undernutrition in children under five and people over 60 according to regions, Colombia, 2003-2016

As for the mortality by region (figure 2), the Atlantic and the Orinoquia-Amazonia regions registered the higher mortality risk for children under five in the two periods. In the case of those over 60, the Atlantic, Oriental, Pacific, and Orinoquia-Amazonia regions showed the highest risks in both periods and the smallest decreases over time.

**Figure 3:**
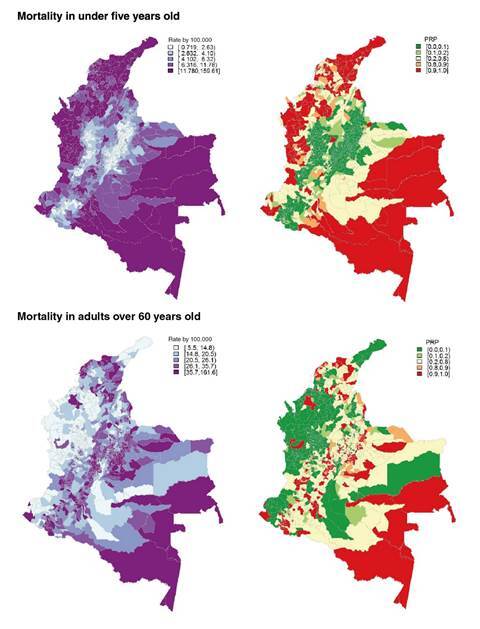
Distribution of smoothed mortality rate (SMR) per 100,000 for undernutrition from 2003 to 2016) in children under five and adults over 60. Next to each mortality map there is a map with the probability of the SMR being above the national rate (PrP). The red color indicates a probability of 90-100% that the SMR is higher than the Colombian rate and the green color indicates the same probability that it is lower.

Figure 3 displays the smoothed mortality rates (SMR) by municipality for the whole period. The areas with the highest risk for children under five were located in the north of the country within the Guajira, Cesar, and Magdalena subregions followed by Barranquilla (metropolitan area) and the Atlantic and Pacific Coasts. In contrast, the lowest mortality risks were observed in the municipalities of Medellín and Bogotá subregions. In the case of mortality for people over 60, municipalities with the highest risk were located within the Atlantic, Oriental, Pacific, and Orinoquia-Amazonia subregions.

Table 1 describes the distribution of deaths from undernutrition according to the socioeconomic indicators. For children under five, the risk of mortality was higher in municipalities with a higher proportion of population with unsatisfied basic needs, less development, and poorer and worse overall performance evidencing a negative social gradient of undernutrition for this age group. In contrast, for those over 60 the social gradient of undernutrition was positive with higher risks of mortality in municipalities with better economic advantages and development (table 2).

**Table 1:** Mortality rate (per 100,000) due to undernutrition in individuals under five years of age and over 60 according to municipal socioeconomic indicators, Colombia, 2003-2016

	Under five years		Over 60 years	
	N (5,804)	RR CI (95%)	N (14,974)	RR CI (95%)
Percentage of the municipal population with unsatisfied basic needs (UBN)				
Richer	1211	3.92 (3.70 - 4.14)	8437	21.8 (21.4 - 22.3)
Quintile 2	903	11.0 (10.3 - 11.8)	2510	32.4 (31.1 - 33.7)
Quintile 3	742	12.4 (11.5 - 13.3)	1594	30.6 (29.1 - 32.2)
Quintile 4	1308	18.1 (17.1 - 19.0)	1402	25.4 (24.1 - 26.7)
Poorer	1625	20.1 (19.1 - 21.1)	1011	18.9 (17.8 - 20.1)
Municipal-level human development index (HDIm)				
Less development	1530	20.0 (19.0 - 21.0)	878	17.4 (16.2 - 18.5)
Quintile 2	918	13.7 (12.8 - 14.6)	1518	26.8 (25.5 - 28.2)
Quintile 3	927	15.4 (14.4 - 16.4)	1346	25.7 (24.3 - 27.1)
Quintile 4	718	10.6 (9.87 - 11.4)	1832	28.1 (26.8 - 29.3)
More development	1661	4.99 (4.75 - 5.23)	9368	23.4 (23.0 - 23.9)
Multidimensional poverty index (MPI)				
Poorer	1343	16.8 (15.9 - 17.7)	937	18.3 (17.1 - 19.4)
Quintile 2	942	15.0 (14.1 - 16.0)	1255	25.2 (23.8 - 26.6)
Quintile 3	951	14.9 (13.9 - 15.8)	1641	30.0 (28.6 - 31.5)
Quintile 4	467	8.19 (7.45 - 8.94)	1600	28.2 (26.9 - 29.6)
Richer	1500	4.41 (4.18 - 4.63)	9854	23.9 (23.4 - 24.4)
Comprehensive performance index				
Outstanding/ Satisfactory	699	3.10 (2.87 - 3.33)	5356	19.2 (18.7 - 19.7)
Medium	1397	8.30 (7.87 - 8.74)	5305	31.6 (30.7 - 32.4)
Low	2060	13.3 (12.8 - 13.9)	3747	26.7 (25.9 - 27.6)
Critical	1047	18.7 (17.6 - 19.9)	879	23.3 (21.7 - 24.8)
Dynamics of the index of water quality for human consumption-IRC				
Getting better	1066	12.8 (12.1 - 13.6)	1873	26.8 (25.6 - 28.0)
Positive constant	1668	8.29 (7.89 - 8.69)	5288	23.9 (23.3 - 24.6)
Negative constant	1560	7.07 (6.72 - 7.42)	5343	23.3 (22.6 - 23.9)
Worse	297	8.54 (7.57 - 9.51)	856	23.5 (21.9 - 25.0)

RR: Relative risk

CI (95%): 95% confidence interval

**Table 2:** Regression mortality risk due to undernutrition in individuals under five and over 60 according to municipal socioeconomic indicators, Colombia, 2003-2016

	Under five years old	Over 60 years old
	RR (95% CI)	RR (95% CI)
Percentage of the municipal population with unsatisfied basic needs (UBN)		
Richer	1.00	1.00
Quintile 2	1.43 (1.11 - 1.80)	0.97 (0.84 - 1.10)
Quintile 3	1.56 (1.19 - 2.01)	0.93 (0.80 - 1.08)
Quintile 4	2.09 (1.60 - 2.68)	0.82 (0.70 - 0.96)
Poorer	2.12 (1.60 - 2.75)	0.63 (0.52 - 0.74)
Municipal-level human development index (HDIm)		
Less development	1.00	1.00
Quintile 2	0.80 (0.66 - 0.96)	1.45 (1.26 - 1.67)
Quintile 3	0.76 (0.61 - 0.93)	1.50 (1.29 - 1.73)
Quintile 4	0.63 (0.49 - 0.79)	1.52 (1.29 - 1.77)
More development	0.52 (0.41 - 0.66)	1.61 (1.37 - 1.88)
Multidimensional poverty index (MPI)		
Poorer	1.00	1.00
Quintile 2	1.27 (1.00 - 1.60)	0.98 (0.86 - 1.10)
Quintile 3	1.50 (1.17 - 1.89)	0.97 (0.84 - 1.10)
Quintile 4	1.62 (1.27 - 2.04)	0.83 (0.72 - 0.96)
Richer	1.75 (1.35 - 2.23)	0.56 (0.48 - 0.66)
Comprehensive performance index		
Outstanding/ Satisfactory	1.00	1.00
Medium	1.26 (1.03 - 1.54)	1.00 (0.90 - 1.12)
Low	1.41 (1.14 - 1.73)	1.00 (0.88 - 1.12)
Critical	1.42 (1.08 - 1.84)	0.92 (0.76 - 1.10)
Dynamics of the index of water quality for human consumption-IRCA		
Getting better	1.00	1.00
Positive constant	0.96 (0.78 - 1.18)	1.02 (0.89 - 1.16)
Negative constant	0.95 (0.78 - 1.14)	1.05 (0.93 - 1.19)
Worse	0.80 (0.59 - 1.05)	0.96 (0.80 - 1.15)

RR: Relative risk

CI (95%): 95% confidence interval

The results of the ecological regression were consistent with these findings (table 2). According to the results for children under five, the risk of dying from undernutrition is higher in the poorest municipalities, i.e., in areas with more unsatisfied basic needs, less development, critical comprehensive performance, and drinking water quality representing low or medium risk. In these municipalities the risk was approximately 50% higher than in reference municipalities. The risk of mortality from undernutrition in children under five was not statistically associated with water quality.

In contrast, adults over 60 had a higher risk of mortality from undernutrition in more developed affluent municipalities with no water quality risk exceeding significantly that of reference municipalities. This risk was not statistically associated with water quality or the municipality’s comprehensive performance.

## Discussion

In general, we found that the mortality due to undernutrition in Colombia decreased during the 2003-2016 period. Moreover, risk maps revealed substantial geographic variations in the size of the risk with reductions over time. Most importantly, we found an association between mortality caused by undernutrition and the socioeconomic indicators of the municipalities.

Our results highlighted a more significant decrease in the risk of undernutrition-related mortality for children under five than for people over 60. This finding may be explained by the fact that Colombian nutritional policies have focused mainly on the population under five to meet the millennium development goals (MDGs) and the sustainable development goals (SDGs) that include the eradication of hunger, the decrease of child mortality and undernutrition, and the development of food and nutrition surveillance systems ^23^.

However, despite this decreasing trend, there are regions that still show a high risk of child mortality. For example, the high mortality rates for children under five in the municipalities of the Atlantic region could be explained partly by the presence of the large mining and energy companies that have caused a decrease in food production resulting from the lack of water sources used elsewhere ^11^.

In the Orinoquia and Amazonia region, the highest rates of mortality due to undernutrition in children under five could may respond to the fact that this region has the highest rates of chronic (29.5%) and global undernutrition (7.5%) in this age group compared to the rest of the country (13.2 and 3.4%, respectively) ^24^. Besides, this region has the highest percentage of indigenous communities whose food production and eating patterns have changed to the detriment of their identity and culture resulting in the loss of their food sovereignty and the substitution of native foods for products that must be purchased ^25^ thus limiting food availability due to their low income levels ^26^.

On the other hand, although all regions have decreased their undernutrition- related mortality rates in children under five, the poorest municipalities continue to have the highest rates. Consistent with the literature review, some of the social conditions associated with these higher mortality rates are the lack of potable water, the scarce institutional infrastructure to carry out social and health programs, the low social security coverage, and the difficulties in food distribution and the supply chain ^27^. In terms of public policies, this situation highlights the need to develop specific strategies to close social gaps.

The situation regarding mortality in adults over 60 is different as mortality rates have decreased very little and are higher in more developed and affluent municipalities, which evidences that, due to different factors such as limited income, this population group does not have adequate access to food, which impacts their health status and those factors that make them more vulnerable in this stage of life ^28^^-^^30^. The lower mortality rates in the poorest municipalities would respond to their location in rural areas where there is a greater availability of food from family farming, the breeding of small animals, self-sufficiency, and family support for obtaining food ^31^^,^^32^.

The risk of mortality due to undernutrition in the urban population over 60 years of age is a global challenge because many of the world’s largest cities are in the least developed countries where the aging process is not occurring slowly and families or states do not have the resources to respond in contrast with the situation in industrialized countries. In these urban centers, inequalities regarding the health conditions of older adults are evident. In Colombia, for example, according to the 2015 National Survey on Health, Welfare, and Aging (*Salud, Bienestar y Envejecimiento,* SABE), eight out of 10 older adults live in urban areas (78.1%) and of the total population surveyed, 30% do not receive assistance from the state or the family ^33^.

In conclusion, the mortality from undernutrition in children under five is an unmet objective and remains a social issue because the rates continue to be high, especially in the poorest municipalities. Simultaneously, the mortality from undernutrition in adults over 60, especially in city residents, constitutes a huge challenge due to the accelerated growth of urban populations in the developing world and their aging process.

Regarding the data quality, a special consideration must be made. Studies on death cause registration in Colombia have shown that this process has improved considerably in the last 30 years and continues to improve ^34^. Cendales, *et al.*^34^ state that: “It is possible that the classification of Colombia rises to the category *country with high quality* in the certification of mortality since our percentage of deaths certified as signs, symptoms, and ill- defined conditions is less than 10%. Likewise, the last WHO coverage report of death shows that Colombia’s coverage rose from 79.9% in the period 1990- 1994 to 88.1% in 1995-1999, 93.1% in the period 2000-2004, and 98.5% in 2009”. Thus, we assumed the data used in our study had enough quality.

An initial review of the data showed that 40% of municipalities consistently reported zero cases of undernutrition in children under five, whereas 9% of the municipalities reported zero cases of mortality in adults over 60. The present analysis assumed that all zero counts represented a true absence of cases in the year analyzed. To improve the accuracy of the municipal estimates, the mortality and population counts were aggregated in a single period (2003-2016). In this way, undernutrition rates were estimated for each of the 644 municipalities that reported at least one case of mortality in children under five and for the 992 municipalities reporting at least one case of mortality in adults over 60. Besides, we used the Bayesian model, which averages the data of neighboring municipalities conservatively decreasing the possible quality/under-registration problems of some municipalities.

On the contrary, as this is an ecological study, our findings can only contribute to the evidence of a possible association between inequality in malnutrition and urban territory conditions, but we cannot affirm that this is necessarily a causal relationship. Additionally, although this design allowed us to detect and evaluate socioeconomic inequalities in malnutrition, it was not possible to identify whether inequalities in the risk of malnutrition among municipalities were due largely to the characteristics of the municipalities (contextual effect) or to the differences between the individuals residing in them (compositional effect). This design does not allow for evaluating the role of individual conditions such as confounders, mediators, or modifiers of the effect of the municipality. In this sense, the ecological relationships we found could be further explored with multilevel analyses taking into account the exploration of both individual and contextual factors.

Finally, we stress the importance of extrapolating our results exclusively at the area level. Given the aggregated nature of the data, it would be erroneous to assume that the statistical association between the socioeconomic variables at the municipal level would be equal to the association between the corresponding variables at the individual level. A multilevel analysis would be the next step in this research to properly explore both individual and municipal-level relationships.
